# Blood flow restriction exercise during microgravity exposure in parabolic flight

**DOI:** 10.1113/EP092767

**Published:** 2025-08-06

**Authors:** Yannick Laflamme, Etienne Chassé, Luke Hughes

**Affiliations:** ^1^ Operational Space Medicine Canadian Space Agency, Government of Canada Quebec Canada; ^2^ Canadian Forces Morale and Welfare Services Ottawa Canada; ^3^ Department of Sport, Exercise and Rehabilitation, Faculty of Health and Life Sciences Northumbria University Newcastle Upon‐Tyne UK

**Keywords:** blood flow restriction, exercise, microgravity, parabola, spaceflight

## Abstract

This case report evaluates whether it is possible to perform blood flow restriction (BFR) exercise during exposure to microgravity. The objectives were three‐fold: (1) to determine if a personalised tourniquet system (PTS) hardware technology performs nominally and enables BFR exercise in microgravity; (2) to determine if BFR augments the exercise stimulus in microgravity in a similar manner to its application on Earth; and (3) to evaluate tolerance and acceptability of performing BFR exercise and operating the PTS hardware in microgravity. Two participants performed resistance squat and deadlift exercises on a flywheel device (inertia of 0.01 kg m^2^) with and without BFR during microgravity in parabolic flight onboard the National Research Council's Falcon 20 aircraft. Heart rate, perceived exertion and discomfort, and the participants’ tolerance and acceptability of performing BFR exercise in microgravity compared to exercise without BFR were measured. Performance of the PTS hardware technology was also evaluated. This case report demonstrates, for the first time, that it is possible to perform BFR exercise in microgravity in a manner that may augment the physiological stress of exercise in an acceptable and tolerable fashion. Importantly, the BFR hardware technology required to perform BFR exercise in an accurate, safe and effective manner performs nominally in microgravity. Future research should aim to conduct investigations during longer exposures to microgravity (i.e. during 3‐ to 6‐month missions on the International Space Station), providing a more comprehensive evaluation of the physiological stimulus provided and the tolerance and acceptability when performing BFR exercise in the space environment.

## Abstract (highlights)

- **What is the main observation in this case?**
  It is possible to perform blood flow restriction (BFR) exercise in microgravity in a manner that may augment the physiological stress of exercise in an acceptable and tolerable fashion. The BFR hardware technology required to perform BFR exercise in an accurate, safe and effective manner performs nominally in microgravity.
- **What insights does it reveal?**
  This will support subsequent research exploring the use of BFR to optimise the current exercise countermeasure programme, and to provide a new modality of exercise for exploration‐class missions.

## 1 INTRODUCTION

It has long been understood that the space environment presents a major challenge to human health. Spaceflight causes deconditioning of several physiological systems, particularly the muscular and cardiovascular systems, which is detrimental to crew health and performance and a critical risk to mission success (Hughes et al., 2022). Exercise is a key countermeasure used to mitigate physiological deconditioning and improve crew health during spaceflight missions (Scott et al., 2019). Our current understanding of the importance of exercise in crew health maintenance is underpinned by decades of physiological research conducted in low Earth orbit and terrestrial spaceflight analogues (e.g. bedrest), which has served to refine exercise protocols (Scott et al., 2019). Recent International Space Station (ISS) data suggest crewmembers now only experience moderate levels of deconditioning; however, there is significant individual variation (Fernandez‐Gonzalo et al., 2021) with some crewmembers experiencing exacerbated physiological changes despite significant investment of time and effort into in‐flight exercise, suggesting that exercise countermeasures are yet to be optimised for all individuals (Scott et al., 2020). As we progress towards exploration‐class missions to Lunar Gateway and beyond, it is anticipated that such missions will involve more physically demanding extravehicular activities (e.g., surface operations) alongside greater physiological deconditioning, increasing reliance on in‐flight exercise to ensure crew health throughout the missions, which is indispensable for mission success. However, these missions will place operational, technical and logistical constraints upon the use of exercise, with less mass, volume, power and time available for exercise and associated hardware (Laws et al., 2020; Scott et al., 2019) within next generation exploration‐class habitats and vehicles such as Orion, Lunar Gateway and private stations such as Vast's Haven 1. National agencies and private space exploration companies are allocating significant resources to the investigation and development of novel exercise devices and technologies that can specifically reduce the exercise load, intensity, time and hardware needed to maintain crew health and performance (Scott et al., 2020). One such approach is flywheel exercise, which has previously been shown to attenuate some aspects of physiological deconditioning during bedrest (Alkner & Tesch, 2004; Irimia et al., 2017; Rittweger et al., 2005).

‘Blood flow restriction’ (BFR) exercise has been identified as having great potential to facilitate maintenance of astronaut health during spaceflight missions by reducing the intensity, duration and hardware required for in‐flight exercise (Behringer & Willberg, 2019; Hughes et al., 2022; Scott et al., 2020). This technique uses a personalised tourniquet system (PTS) and pneumatic tourniquet cuff applied proximally on the limb(s) which is inflated to a pre‐determined pressure to compress limb vasculature (Hughes et al., 2018). The goal is to partially restrict arterial inflow and completely restrict venous outflow in tissues distal to the tourniquet cuff, creating a hypoxic environment that stimulates adaptive responses (Patterson et al., 2019). Applying BFR during exercise characterised by low external load alters local and systemic tissue physiology (Franz et al., 2020), exacerbating acute psychophysiological responses to exercise (Bielitzki et al., 2024). The ischaemic and hypoxic environment accelerates fatigue, triggering a cascade of mechanistic pathways (Patterson et al., 2019; Pearson & Hussain, 2015) which, when applied repeatedly as a training intervention, elicits superior adaptations in the muscle, cardiopulmonary and vascular systems when compared with external load‐ or intensity‐matched exercise (Patterson et al., 2019). Critically, these adaptations have been found to be comparable to those achieved with high‐intensity exercise (Grønfeldt et al., 2020; Mouser et al., 2019). BFR exercise requires minimal exercise hardware/resistance, and uses lower intensities and shorter durations of exercise, and thus has the potential to: (1) optimise the current ISS exercise countermeasure programme, and (2) provide a method to maintain crew health within the constraints of exploration‐class missions (Behringer & Willberg, 2019; Hughes et al., 2022; Scott et al., 2020). Furthermore, in the event that flywheel exercise training causes too much stress on the Orion vehicle, BFR could potentially be used to reduce the external exercise load while maintaining internal exercise intensity.

While BFR exercise is a validated method of improving exercise efficiency in ground studies (Hughes et al., 2022), its viability and efficacy have not yet been demonstrated in space. Furthermore, recent data suggest that a reduced gravitational environment, achieved via horizontal human suspension, affects automatic measurement of limb occlusion pressure (LOP) by increasing the applied pressure required to fully occlude the limb (Swain et al., 2024), which is required to individualise the BFR stimulus (Patterson et al., 2019). If BFR exercise is to be explored in the space environment, it is critical to first determine if BFR exercise and associated hardware technology perform nominally in microgravity, that is, via parabolic flight, which offers the opportunity to conduct research during short, repeated exposures to microgravity without the constraints of terrestrial analogue systems (e.g. horizontal suspension). Therefore, the key objectives of this case report were three‐fold: (1) to determine if the PTS hardware technology performs nominally and enables BFR exercise in microgravity; (2) to determine if BFR augments the exercise stimulus in microgravity in a similar manner to its application on Earth; and (3) to evaluate tolerance and acceptability of performing BFR exercise and operating the PTS hardware in microgravity.

## 2 METHODS

### 2.1 Participants

Two healthy males, both of whom had previous experience of parabolic flight, participated in this study (participant 1: 32 years, 174 cm, 91.4 kg; participant 2: 31 years, 178 cm, 89.0 kg). Both participants were free from any cardiovascular, pulmonary and metabolic diseases, and musculoskeletal injuries in the preceding 12 months, and did not present with any contraindications to BFR exercise and tourniquet use. Additionally, they refrained from strenuous exercise, caffeine and alcohol in the 24 h prior to data collection. Ethical approval was granted by the National Research Council Canada Human Research Ethics Board (2019‐122 under Generic: 2016‐34) and conformed to the Declaration of Helsinki.

### 2.2 Protocol

This case study was conducted as part of a larger parabolic flight campaign examining the feasibility of performing flywheel exercise in microgravity. For the BFR exercise experiment, participants performed squat and deadlift exercise on a flywheel‐enabled exercise device (K‐Box Lite, Exxentric, Stockholm, Sweden) both with and without BFR. Flywheel device exercise intensity is determined by the user's force and speed, offering unlimited resistance throughout the motion. Unlike traditional weight training, resistance depends on user tempo of movement (cadence) rather than just the load (National Strength & Conditioning Association, 2024).

**FIGURE 1:**
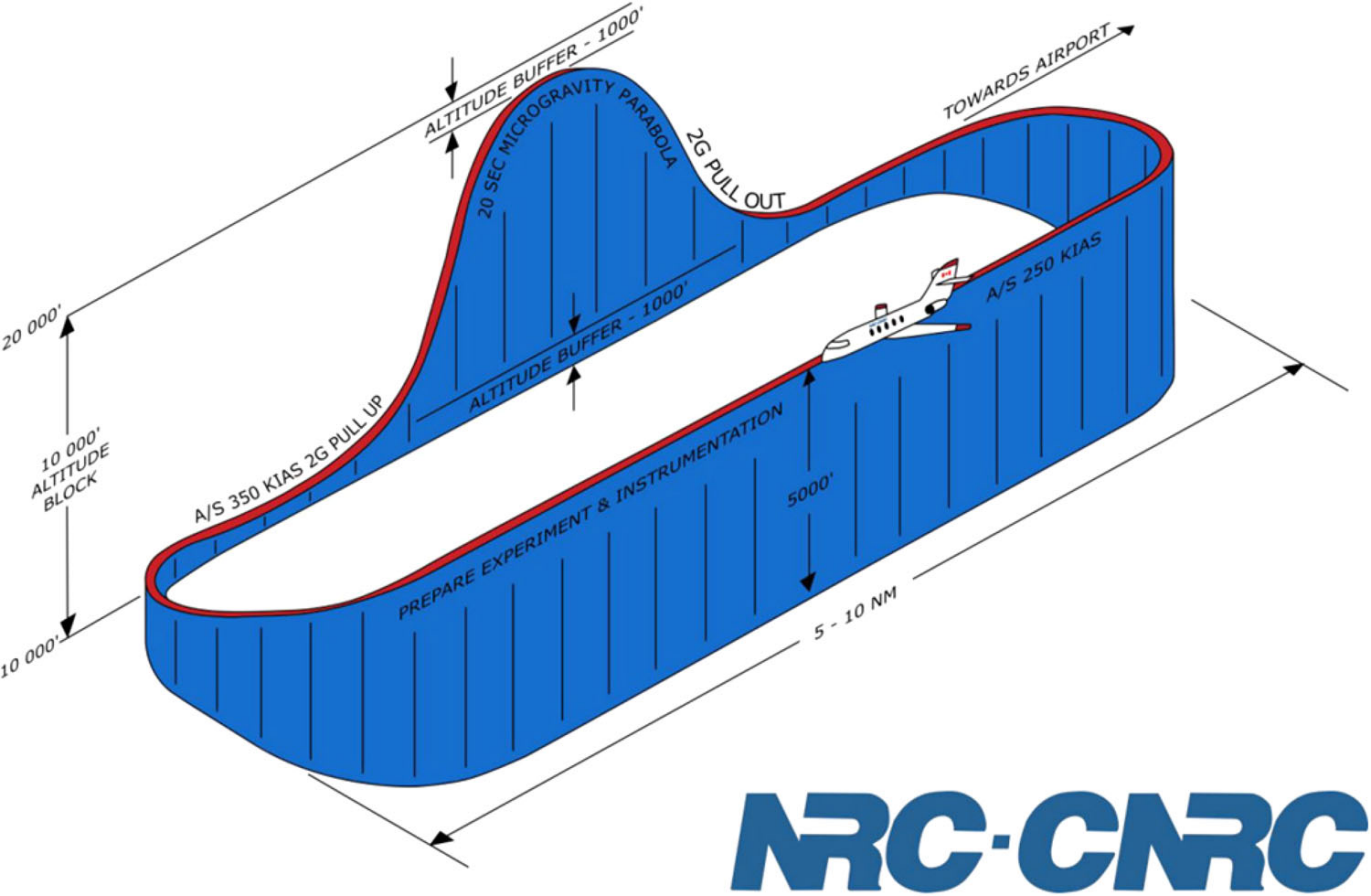
Overview of gravity exposures during parabolic flight. A/S, airspeed; KIAS, knots indicated airspeed; NRC‐CNRC, National Research Council Canada.

Participants performed four blocks of four parabolas with approximately 5–10 min between blocks on the National Research Council of Canada's Falcon 20 aircraft. Each parabola provided approximately 18–22 s of microgravity (Figure 1), with approximately 40 s rest between parabolas (Table 1). Participants only exercised during exposures to microgravity, and were instructed to perform as many repetitions as possible in a safe and controlled manner.

**TABLE 1:** Overview of protocol.

**Parabola**	**Duration**	**Rest**	**Exercise**	**Resistance**	**BFR**
1–2	18–22 s	40 s	Familiarization	0.01 kg m^2^	No
3–6			Squat		No
7–10			Squat		Yes
11–14			Deadlift		No
15–18			Deadlift		Yes

Abbreviation: BFR, blood flow restriction.

**FIGURE 2:**
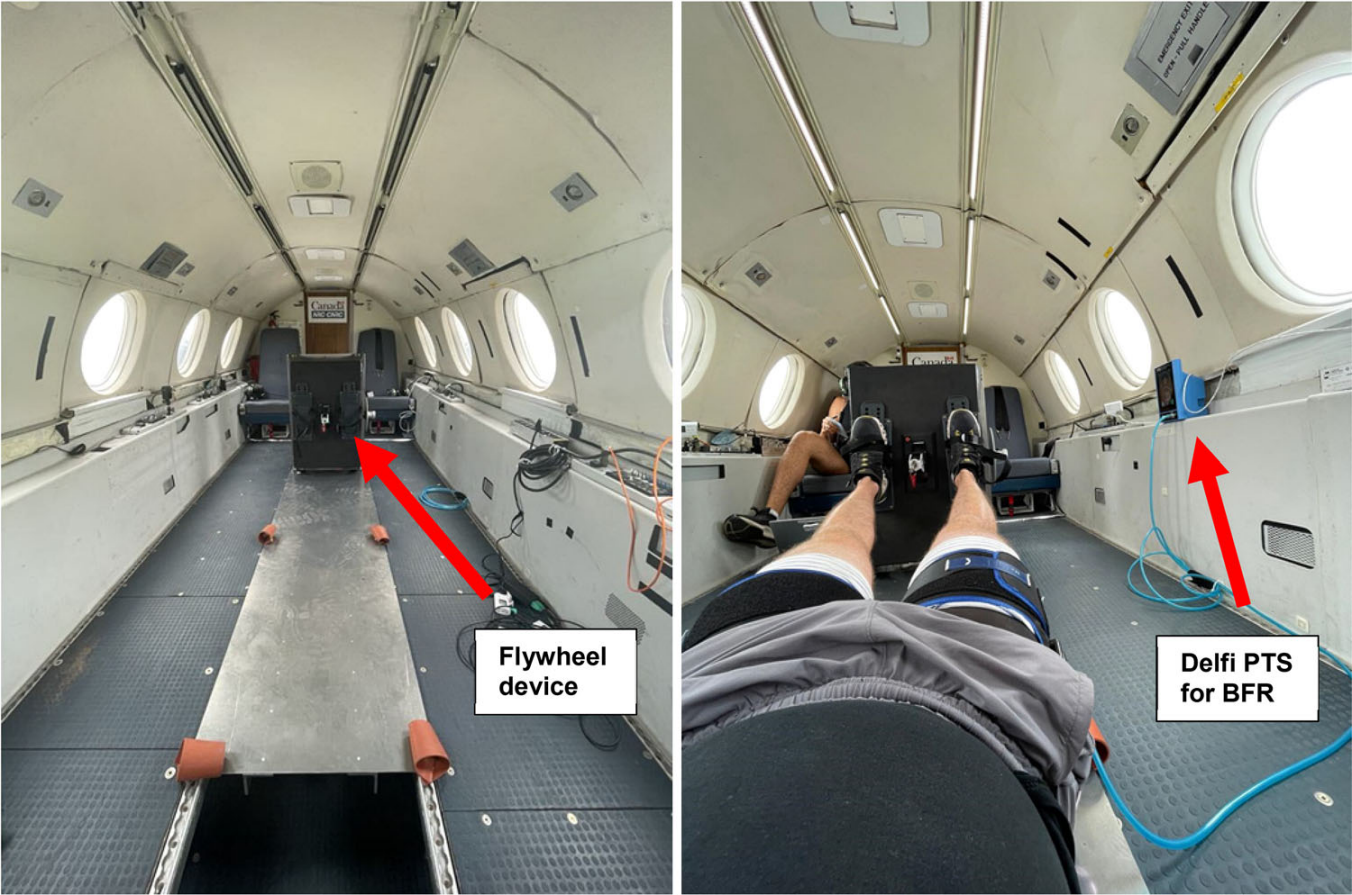
In‐flight blood flow restriction exercise setup on NRC's Falcon 20 aircraft. BFR, blood flow restriction; PTS, personalised tourniquet system.

An automatic Personalised Tourniquet System (Delfi Medical Inc., Vancouver, BC, Canada) was used to perform BFR. This system has a dual‐purpose tourniquet cuff (11.5 × 86 cm) connected by airtight hose tubing to a personalized tourniquet device. The commercially available device enables unilateral BFR application (i.e. one limb per device). Due to the need for efficient application of BFR, and considering that future BFR applications in a space environment would most likely be bilateral in nature (Hughes et al., 2022), a second tourniquet cuff was placed proximally on the contralateral lower limb and connected to the first cuff via airtight hose tubing to create a ‘daisy‐chain’ of airflow, thus enabling bilateral BFR exercise to be performed (Figure 2). This system automatically measures ‘limb occlusion pressure’ (LOP), defined as the minimum pressure required for complete arterial occlusion in a limb (McEwen et al., 2015), and regulates pressure during exercise within clinically acceptable limits (Mouser et al., 2019). This system has been validated against Doppler ultrasound and distal photoplethysmography techniques for measuring LOP (Hughes & McEwen, 2021; Masri et al., 2016) and is reliable on a test–retest basis in ground (Hughes et al., 2018) and space analogue studies (i.e. horizontal suspension and body tilt) (Swain et al., 2024). Current recommendations are to inflate the BFR tourniquet cuff to a target pressure of between 40% and 80% LOP (Patterson et al., 2019), with higher pressures potentially eliciting greater muscular and vascular adaptations when implemented as a training intervention (Lixandrão et al., 2015; Mouser et al., 2019). As this was the first attempt at performing BFR exercise in microgravity, target pressure was set to 60% LOP. The LOP measurement was performed in‐flight (Swain et al., 2024) in the body position that the participants would exercise (Hughes et al., 2018) to ensure accurate prescription of the target pressure. For the BFR trials, the bilateral BFR tourniquet cuffs were inflated to 60% LOP at 15 s prior to the first parabola and remained inflated continuously throughout each block of parabolas and deflated following the final parabola of each block.

### 2.3 Outcome measurements

The following data was collected:

1. Limb occlusion pressure: measured prior to the first block of parabolas, to enable BFR exercise and determine if the PTS hardware technology performed nominally in microgravity (objective 1)
2. Heart rate (bpm): measured continuously during each block of parabolas using the H10 sensor (Polar, Kempele, Finland), with maximum heart rate for each set of exercise determined, to determine if application of BFR augments low intensity exercise in microgravity (objective 2)
3. Number of repetitions: monitored and recorded for each parabola, to determine if BFR application impacts exercise performance (objective 2).
4. Perceived discomfort, exertion and acceptability: participants' perception of discomfort and exertion when performing the exercises with BFR versus without, and their opinion on whether a BFR exercise programme would be acceptable to perform in microgravity, was recorded using a bespoke survey (objective 3). The following questions were asked:
  - When performing exercises with BFR, how did the discomfort compare to that experienced for the same exercises without BFR?
  - When performing exercises with BFR, how did your perceived exertion compared to that experienced for the same exercises without BFR?
  - Do you think it would be acceptable to perform BFR exercise as part of a countermeasure programme?

### 2.4 Data analysis

Data are presented as means ± SD unless stated otherwise. LOP, target pressure, total time inflated, rest time, maximum heart rate (HR_max_) and number of repetitions for each set of exercise are presented in Tables 2, 3 and 4.

**TABLE 2:** Limb occlusion and target BFR pressures (*n* = 2).

	**Participant 1**	**Participant 2**
LOP (mmHg)	247	222
Target pressure (mmHg)	148	133
Total time inflated (min)	6	6
Rest time (s)	60	60

Abbreviations: BFR, blood flow restriction; LOP, limb occlusion pressure.

## 3 RESULTS

Both participants completed all parabolas with no adverse events. The PTS for BFR performed nominally in microgravity and was able to successfully perform automatic measurement of limb occlusion pressure without error, and thus it was possible to perform BFR exercise (see videos provided as Supporting information, Supplements 1 and 2). The data concerning BFR prescription and application are presented in Table 2. Heart rate appeared to be elevated during parabolas where exercise was performed with BFR versus without in both participants (Table 3). The number of repetitions completed remained similar when exercise was performed with BFR versus without (Table 3). Both participants reported that perceived discomfort in the lower limbs and overall perceived exertion tended to be greater when exercise was performed with BFR versus without; importantly, this was deemed as tolerable. Finally, both participants reported that they felt it is feasible to perform BFR exercise in microgravity during parabolic flight. Participants’ specific answers to the questions can be found in Table 4.

**TABLE 3:** HR_max_ and repetitions completed.

	Set 1	Set 2	Set 3	Set 4	Mean diff.
Participant 1					
HR_max_ (bpm^−1^)					
Squat					
BFR	128	130	133	137	8 ± 4
No BFR	125	119	128	125	
Deadlift					
BFR	142	151	147	152	7 ± 5
No BFR	140	137	143	146	
Repetitions completed					
Squat					
BFR	8	15	15	14	−1 ± 3
No BFR	13	14	13	15	
Deadlift					
BFR	16	15	15	15	2 ± 2
No BFR	12	12	15	16	
Participant 2					
HR_max_ (bpm^−1^)					
Squat					
BFR	107	111	116	114	4 ± 2
No BFR	105	108	110	111	
Deadlift					
BFR	115	118	115	118	5 ± 3
No BFR	107	112	113	116	
Repetitions completed					
Squat					
BFR	8	13	12	14	0 ± 2
No BFR	7	14	14	12	
Deadlift					
BFR	13	12	12	14	0 ± 2
No BFR	11	11	13	16	

Data are presented as means ± SD (*n* = 2). Of note, the squat exercise tends to be unstable when completed in micro‐gravity with the flywheel device and this explains the lower repetition count from set 1 of participants 1 and 2. BFR, blood flow restriction; bpm, beats per minute; HR, heart rate.

**TABLE 4:** Specific responses to qualitative question.

**Question: When performing the exercises with BFR, how did the discomfort compare to that experienced for the same exercises without BFR?**	
Participant 1	It was not uncomfortable, but could definitely feel that they were on. The muscular burn started to feel apparent especially during squats and mostly due to the fact that we were resting in a contracted position
Participant 2	A little discomfort from the cuff but very manageable

## 4 DISCUSSION

The overarching objectives of this case report were to determine if the PTS hardware technology performs nominally in microgravity to enable BFR exercise to be performed in a tolerable and acceptable manner. It was found that:

(1) The PTS hardware technology was able to perform automatic LOP measurement and control pressure during the microgravity portions of flight.
(2) Performing BFR during low intensity exercise in microgravity exacerbated heart rate response.
(3) Performing BFR during low intensity exercise in microgravity was tolerable and acceptable to perform.

To safely and effectively prescribe BFR exercise, the ability to accurately measure LOP is critical for the individualisation of the BFR stimulus (Hughes et al., 2024). Terrestrial research has demonstrated that body position (Hughes et al., 2018; Sieljacks et al., 2018), tilt angle (Swain et al., 2024) and reduced axial gravitational loading (Swain et al., 2024) can impact the pressure required to fully occlude arterial blood flow in a limb (i.e. limb occlusion pressure). This is thought to be caused by postural related changes in peripheral blood flow and venous blood pooling (Hughes et al., 2018; Sieljacks et al., 2018; Swain et al., 2024). Indeed, several cardiovascular parameters (e.g. stroke volume and systemic peripheral resistance) are sensitive to head‐down and head‐up tilt manoeuvres where the gravitational vector acting along the body is manipulated (Whittle et al., 2022). As blood pressure, flow and regulatory mechanisms behave differently in microgravity (Norsk, 2014; Norsk et al., 2015), it is conceivable that BFR devices designed to automatically measure LOP and maintain and regulate cuff pressure close to the target pressure, might not operate nominally in microgravity. This would present a major barrier to efforts to develop BFR exercise as a spaceflight countermeasure through in‐flight investigations. This case report is the first to demonstrate that the PTS for BFR operates nominally in microgravity, which lends support to future work.

This case report is the first to demonstrate that application of BFR during low intensity exercise exacerbates the heart rate and perceptual response to exercise, as evidenced by elevated heart rate and perceived exertion and discomfort, in microgravity in a similar manner to that observed on Earth. This provides promise for future research investigating the potential for using BFR to improve exercise efficiency and crew health in the space environment. Importantly, the anticipated elevated discomfort during BFR exercise in microgravity was tolerable, and overall BFR exercise was perceived to be acceptable to perform in this context.

It is clear that this case report is not without its limitations. In addition to the small sample size, it was not possible to prescribe individualised exercise load relative to each participant's maximum strength, and specific exercise loading intensity could not be determined, owing to the flywheel exercise hardware used. Due to logistical constraints of the flight, it was not possible to randomise the order of the sessions for each participant. Furthermore, only rudimentary measures of the physiological impact of exercise in microgravity were possible within the constraints of this flight. While parabolic flights provide the opportunity to conduct exercise countermeasure research in microgravity, it only offers a short exposure (i.e. 18–22 s) within which to collect data. Nevertheless, our preliminary data suggest that even a short burst of BFR exercise in microgravity is feasible to perform, which provides promise for future in‐flight research where BFR exercise can be performed for longer durations with sophisticated physiological measurement equipment (i.e. on the ISS).

### 4.1 Conclusion and perspectives

This case report demonstrates, for the first time, that it is possible to perform BFR exercise in microgravity. Importantly, the BFR hardware technology required to perform BFR exercise in an accurate, safe and effective manner performed nominally in microgravity. Future research should aim to conduct investigations during longer exposures to microgravity (i.e. during 3–6 month missions on the ISS), providing a more comprehensive evaluation of the physiological stimulus provided. Eventually, perhaps in the not‐to‐distant future, it will be necessary to implement an in‐flight BFR training programme in crewmembers, to evaluate the efficacy of this novel exercise countermeasure to potentially mitigate deconditioning and maintain crew health, as well as crewmember tolerance and acceptability of such an intervention. This will support subsequent research exploring the use of BFR to optimise the current exercise countermeasure programme, and provide a new modality of exercise for exploration‐class missions.

## AUTHOR CONTRIBUTIONS

Conception of the project: Yannick Laflamme, Etienne Chassé and Luke Hughes. Data collection: Yannick Laflamme and Etienne Chassé. Data analyses: Etienne Chassé, Yannick Laflamme and Luke Hughes. Data interpretation: Yannick Laflamme, Etienne Chassé and Luke Hughes. Manuscript writing: Yannick Laflamme, Etienne Chassé and Luke Hughes. All authors have read and approved the final version of this manuscript and agree to be accountable for all aspects of the work in ensuring that questions related to the accuracy or integrity of any part of the work are appropriately investigated and resolved. All persons designated as authors qualify for authorship, and all those who qualify for authorship are listed.

## CONFLICT OF INTEREST

L.H. acts in a scientific advisory capacity and delivers educational content for Delfi Medical Innovations Inc.

## Supporting information



Supplement 1 and 2. Videos of BFR exercise being performed during microgravity in parabolic flight.

## Data Availability

All individual data for all participants are provided in Tables 2 and 3. Further details can be obtained from the corresponding author upon reasonable request.
